# Kidney Biopsy Findings After Lung Transplantation

**DOI:** 10.1016/j.ekir.2024.07.005

**Published:** 2024-07-06

**Authors:** David de Saint Gilles, Marion Rabant, Aurélie Sannier, Charlotte Mussini, Alexandre Hertig, Antoine Roux, Alexandre Karras, Eric Daugas, Vincent Bunel, Jerome Le Pavec, Renaud Snanoudj

**Affiliations:** 1 Nephrology and Transplantation Department, Bicêtre Hospital, Assistance Publique-Hôpitaux de Paris, Université de Paris Saclay, Le Kremlin-Bicêtre, France; 2 Pathology Department, Necker Hospital, Assistance Publique-Hôpitaux de Paris, Université de Paris Cité, Paris, France; 3 Pathology Department, Bichat Hospital Claude Bernard, Assistance Publique-Hôpitaux de Paris, Université de Paris Cité, Paris, France; 4 Pathology Department, Bicêtre Hospital, Assistance Publique-Hôpitaux de Paris, Université de Paris Cité, Paris, France; 5 Nephrology Department, Foch Hospital, Université de Versailles Saint-Quentin-en-Yvelines, Suresnes, France; 6 Pneumology Department, Foch Hospital, Université de Versailles Saint-Quentin-en-Yvelines, Suresnes, France; 7 Nephrology Department, European Georges Pompidou Hospital, Assistance Publique-Hôpitaux de Paris, Paris, Université de Paris Cité, France; 8 Nephrology Department, Bichat Claude Bernard Hospital, Assistance Publique-Hôpitaux de Paris, Université de Paris Cité, Paris, France; 9 Pneumology Department, Bichat Claude Bernard Hospital, Assistance Publique-Hôpitaux de Paris, Université de Paris Cité, Paris, France; 10 Pneumology Department, Marie Lannelongue Hospital, Le Plessis-Robinson, France

**Keywords:** acute kidney injury, calcineurin inhibitor toxicity, chronic kidney disease, kidney pathology, lung transplantation, thrombotic microangiopathy

## Abstract

**Introduction:**

The early diagnosis of histological kidney damage after lung transplantation (LT) is of paramount importance given the negative prognostic implications of kidney disease.

**Methods:**

Three pathologists analyzed all kidney biopsies (KBs) (N = 100) performed from 2010 to 2021 on lung transplant patients in 4 Paris transplantation centers.

**Results:**

The main indication for biopsy was chronic renal dysfunction (72% of patients). Biopsies were performed at a median of 26.3 months after transplantation and 15 months after a decline in estimated glomerular filtration rate (eGFR) or the onset of proteinuria. Biopsies revealed a wide spectrum of chronic lesions involving the glomerular, vascular, and tubulointerstitial compartments. The 4 most frequent final diagnoses, observed in 18% to 49% of biopsies, were arteriosclerosis, acute calcineurin inhibitor (CNI) toxicity, thrombotic microangiopathy (TMA) and acute tubular necrosis (ATN). TMA was significantly associated with a combination of mTOR inhibitors (mTORi) or CNIs with biological signs present in only 50% of patients. The eGFR was poorly correlated with most lesions, particularly percent glomerulosclerosis, and with the risk of end-stage renal disease (ESRD). Thirty-four patients progressed to ESRD at an average of 20.1 months after biopsy. Three factors were independently associated with the risk of ESRD: postoperative dialysis, proteinuria >3 g/g and percent glomerulosclerosis >4%.

**Conclusion:**

Given the great diversity of renal lesions observed in lung transplant recipients, early referral to nephrologists for KB should be considered for these patients when they present with signs of kidney disease.

## Graphical abstract

**Figure:**
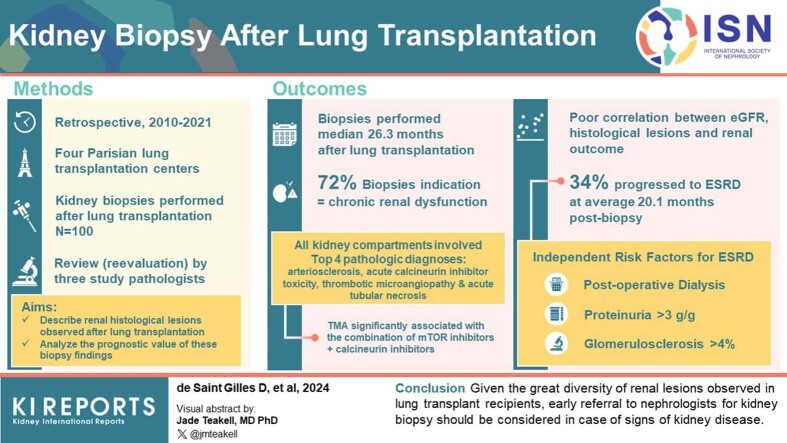

Since its first report in 1963,^1^ the results of LT have continuously improved. In France, with 384 LTs performed in 2019, the current median patient survival is 113 months.^2^ However, medical care for LT recipients is complex because of the wide range of comorbidities and posttransplantation complications.

Renal failure after nonrenal solid organ transplantation is a frequent complication, and in LT recipients, the prevalence of severe chronic kidney disease (CKD) with an eGFR <30 ml/min per 1.73 m^2^ is 15.8% at 5 years.^3^ Regarding acute kidney injury (AKI), a meta-analysis^4^ in unselected LT recipients using several definitions of AKI reported a prevalence of 52%, with dialysis in 9% of patients. Postoperative AKI has multiple^5^ risk factors (pulmonary pathology, intraoperative hemodynamics, duration of mechanical ventilation, use of cardiopulmonary bypass,^6^ etc.) and has a major impact on the early morbidity and mortality of patients.^4^

Preventing AKI and/or CKD if possible is important because their consequences are severe: ESRD^7^ requiring dialysis or kidney transplantation,^8^, ^9^, ^10^ and a 4-fold increased risk of mortality.^3^^,^^11^

Multiple studies have focused on the risk factors for CKD^3^^,^^7^^,^^12^, ^13^, ^14^ after nonrenal organ transplantation, such as pretransplant renal dysfunction, episodes of perioperative AKI, pretransplant diabetes or hypertension, and maintenance immunosuppressive treatment based on CNIs. Indeed, CNIs exhibit significant and polymorphic toxicity, including acute and chronic nephrotoxicity. Since 2010, several studies, including the NOCTET study,^15^, ^16^, ^17^ have suggested as an alternative to classical triple therapy (CNI-mycophenolate-steroids), a quadruple therapy including low-dose CNI, mTORi, steroids, and mycophenolate, aiming to reduce CNI exposure and thus improve long-term renal function. The use of mTORi is also growing in LT because they are thought to preserve lung function and reduce the development of posttransplant bronchiolitis obliterans.^18^^,^^19^

However, a limitation of most studies on CKD occurring after organ transplantation is the lack of histological documentation. Although the prevalence of post-LT CKD is well-documented, only a few small studies have described the renal pathology,^20^^,^^21^ and the largest such series^22^ included 28 patients. The main lesions observed in these studies were CNI toxicity, BK virus nephropathy, TMA, and benign nephrosclerosis.

The aim of our study was to provide an updated comprehensive description of renal histological lesions observed through the reevaluation of all KBs performed over the last 10 years in the 4 Parisian LT centers. We also aimed to analyze the prognostic value of these biopsy findings.

## Methods

### Patients

This French, retrospective, multicenter study included all lung transplant recipients from 4 Parisian transplant centers who had adequate KBs between January 1, 2010, and December 31, 2021. We excluded patients who received another organ combined with the lung and patients who were on dialysis at the time of LT.

All patients provided written consent for the use of their clinical, biological, and histological data before the KB. We used the STROBE cohort reporting guidelines.^23^

### Histological Lesions

We identified 100 patients with an adequate biopsy (at least 7 glomeruli) that were centrally reviewed by 3 kidney pathologists who were unaware of the clinical characteristics of the patients and used a semiquantitative common grid (Supplementary Table S1, based on the Banff 2019 classification of kidney transplant pathology). The following elementary lesions were quantified: interstitial fibrosis and tubular atrophy (IF/TA), inflammation in fibrous areas, inflammation in nonfibrotic areas, total inflammation, tubulitis, arteriolar hyalinosis (ah), vascular fibrous intimal thickening, ATN, the presence of isovolumetric tubular vacuolization, tubular macrovacuolizations and myocyte vacuolization, and the percentage of glomerulosclerosis observed and after correction by age.^24^

These primary lesions allowed us to establish 1 or more final diagnoses. We only screened acute CNI nephrotoxicity (based on moderate to severe isovolumetric tubular vacuolization) because of the lack of specificity of the chronic nephrotoxicity lesions (ah, stripped band fibrosis).

All staining procedures, including hematoxylin-eosin-Saffron, periodic acid-Schiff, Masson’s trichrome, and Jones methenamine silver staining were performed at the time of diagnosis.

### Clinical Data

The clinical data were retrieved from medical files. For each patient, we noted pulmonary disease status and medical history. Diabetes and hypertension were defined by the need for a specific medication. For surgery, we noted whether the patient benefited from a super-emergency procedure, that is, a national priority because of a life-threatening condition. We recorded the immunosuppressive treatment at the time of LT and KB.

Systemic TMA was defined by the presence of biological signs, that is, thrombocytopenia (<150 g/l), low haptoglobin and/or the presence of schistocytes on the blood smear.

All GFR values were estimated by using the CKD-Epidemiology Collaboration 2021 formula or by the SCHWARTZ formula (for patients aged <16 years). The severity of postoperative AKI was graded according to the Kidney Disease: Improving Global Outcomes (KDIGO) classification.^25^ Proteinuria was expressed as the urinary protein-to-creatinine ratio (PCR).

Access to the complete evolution of renal function allowed us to measure the delay between the first biological sign of renal injury (increase in eGFR >20%, except for a resolutive episode of AKI, or a PCR ≥0.5 g/g) and the date of the KB. Patients who underwent biopsy for AKI on CKD were classified as those who underwent biopsy for CKD.

ESRD was defined based on the day of initiation of renal replacement therapy or renal transplantation.

### Statistical Analyses

Continuous variables were described using means and SDs or medians and interquartile ranges. To study associations between histological lesions and clinical parameters, we used Student’s *t* test (or Wilcoxon tests for nonparametric variables) or the χ^2^ test (or Fisher exact test, if appropriate). Correlations between continuous variables were assessed with Pearson or Spearman tests.

Renal survival analysis was performed from LT to dialysis or kidney transplantation using Kaplan‒Meier estimates. Renal survival was censored at a maximal time of 10 years after LT or death for the patients who died before the ERSD.

Univariate and multivariate Cox proportional hazards models were applied using hazard ratios to identify clinical and histological factors associated with the risk of ESRD from the day of KB. The final multivariate Cox model was obtained by entering the risk factors from the univariate model that achieved *P* < 0.10. The proportional hazard assumption was assessed graphically by plotting the Schoenfeld residuals.

We used STATA (version 15.1, StataCorp., College Station, TX) and R (version 4.2.3, R Foundation for Statistical Computing) for the analyses. All the statistical tests were 2-sided, and *P* < 0.05 was considered significant.

## Results

### Characteristics of the Study Population at Lung Transplantation

The patients who had KB represent approximately 3% of the patients transplanted during the study period. For the 100 patients in our study population, the 3 main indications for LT were cystic fibrosis, pulmonary fibrosis, and emphysema, with clear center clustering (Supplementary Table S2). The median age of patients at LT was 40.4 years, and 5 patients were younger than 18 years. The median eGFR was 112 ml/min per 1.73 m^2^, and only 1 patient had an eGFR <60 ml/min per 1.73 m^2^.

Regarding immunosuppressive maintenance treatment, 73% of patients received a combination of steroids, mycophenolate, and tacrolimus.

Postoperatively, 45.5% of the patients experienced an episode of KDIGO stage 2 or 3 AKI. Thirteen patients (13.7%) required transient postoperative dialysis, with the exception of 2 patients who remained on dialysis and eventually died.

### Clinical and Biological Presentation at the Time of KB

**Figure 1:**
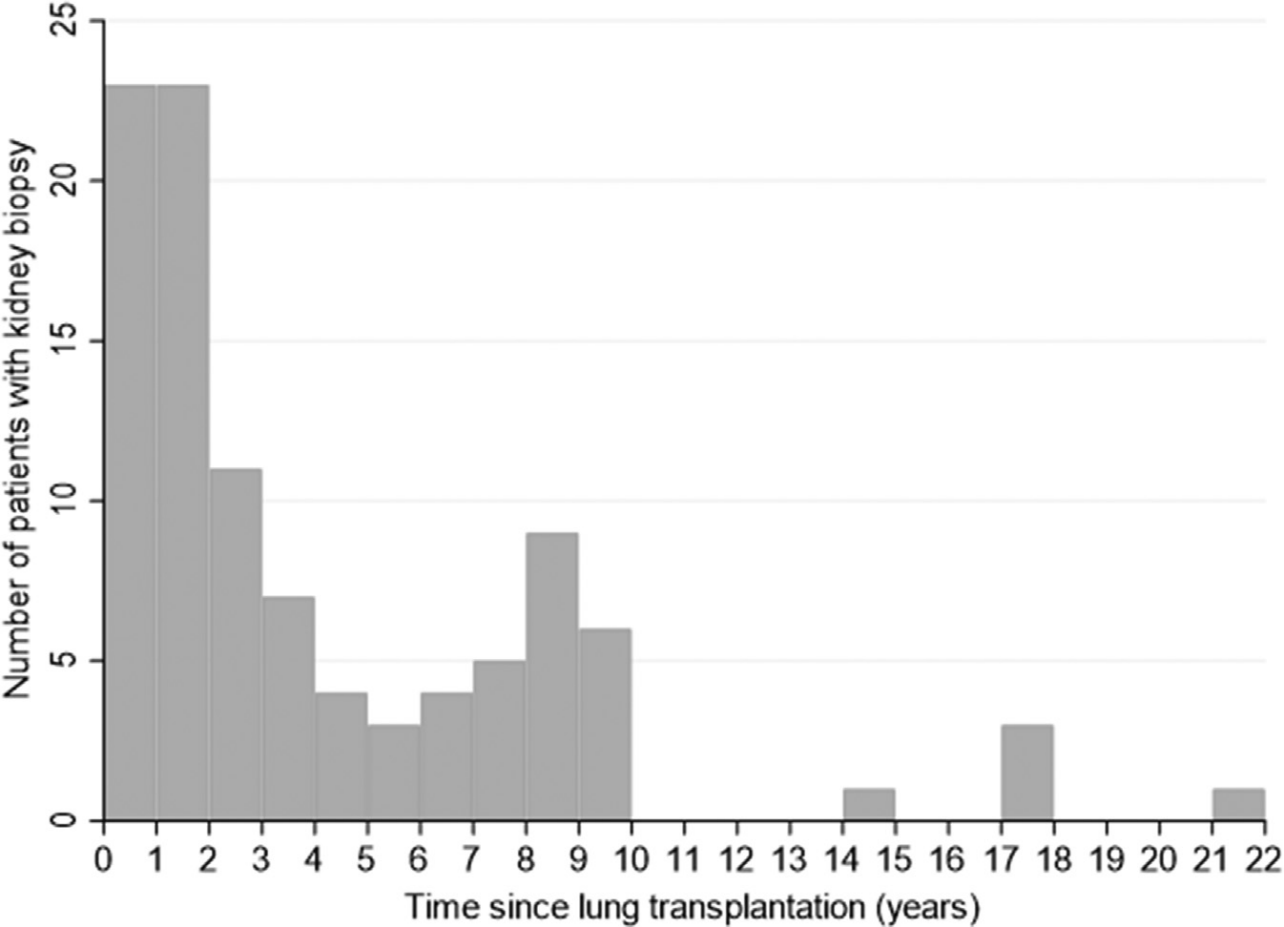
Delay between the lung transplantation and the kidney biopsy. Each chart represents the number of patients who had a renal biopsy over 1 year.

We analyzed a total of 100 KBs performed at a median of 26.3 months after LT, and 5 biopsies were performed more than 10 years post-LT (Figure 1 and Table 1). The frequencies of diabetes and hypertension were greater than those at LT (51.5% and 61.2% vs. 19.6% and 14.3%, respectively). The median delay between the occurrence of biological signs of renal injury (proteinuria or a decrease in the eGFR) and KB was 15 months.

**Table 1:** Characteristics of the population at the time of kidney biopsy (KB)

Clinical parameters	Patients (*N* = 100)	MD
Age (yr), median (IQR)	47.0 (31.7–56.8)	0
BMI, median (IQR)	20.0 (18.1–22.1)	14
Diabetes mellitus, *n* (%)	51 (51.5)	1
Hypertension, *n* (%)	60 (61.2)	2
Time between LT and KB (mo), median (IQR)	26.3 (13.0–85.5)	0
Indication, *n* (%)		0
Acute kidney injury	20 (20)	
Chronic kidney disease	72 (72)	
Isolated proteinuria	4 (4)	
Assessment before lung re-transplantation	4 (4)	
Time since sign of renal injury^a^(mo), median (IQR)	15 (9–36)	1
Kidney function		
eGFR (ml/min per 1.73 m^2^), median (IQR)	32.5 (25–43)	2
Serum creatinine (μmol/l), median (IQR)	185 (149–245)	2
Patient on hemodialysis, *n* (%)	2 (2)	0
PCR (g/g), median (IQR)	0.57 (0.24–2.1)	3
PCR >0.5 g/g, *n* (%)	56 (57.7)	3
Hematuria, *n* (%)	13 (14.8)	12
Systemic TMA, *n* (%)	15 (15)	0
Immunosuppressive treatment		
Maintenance treatment, *n* (%)		0
Steroids	91 (91)	
Tacrolimus	93 (93)	
Cyclosporine	5 (5)	
Mycophenolate	67 (67)	
Azathioprine	9 (9)	
Everolimus	32 (32)	
Rapamune	2 (2)	
Leflunomide	4 (4)	
Tacrolimus trough level (ng/ml), median (IQR)	7.15 (5.2–8.9)	22
Everolimus trough level (ng/ml), median (IQR)	4.5 (4.0–7.3)	10
Combination CNI + mTORi at KB, *n* (%)	32 (32)	0

BMI, body mass index; CNI, calcineurin inhibitor; eGFR: estimated glomerular filtration rate by chronic kidney disease-epidemiology collaboration 2021; IQR, interquartile range; KB, kidney biopsy; LT, lung transplantation; MD, missing data; mTORi, mTOR inhibitor; PCR, urinary protein-to-creatinine ratio; TMA, thrombotic microangiopathy.

a For chronic kidney disease and isolated proteinuria.

Compared to their initial immunosuppression, more patients were treated with an mTORi (32% vs. 2%) in combination with a CNI at the time of biopsy (mainly everolimus and tacrolimus).

The main indication for KB was CKD (72%). eGFR decreased in 96% of patients, from a median value of 112 ml/min per 1.73 m^2^ at LT to 32.5 ml/min per 1.73 m^2^ at KB. In 55% of patients, PCR was greater than ≥ 0.5 g/g (15% in the nephrotic range).

Among the 100 biopsies performed, 15 patients (15%) experienced significant bleeding consisting of macroscopic hematuria in 6 patients and/or perirenal hematoma in 14 patients. Four patients (4%) required a red blood cell transfusion and 2 patients (2%) required an interventional hemostasis procedure.

### Renal Elementary Histological Lesions

**Figure 2:**
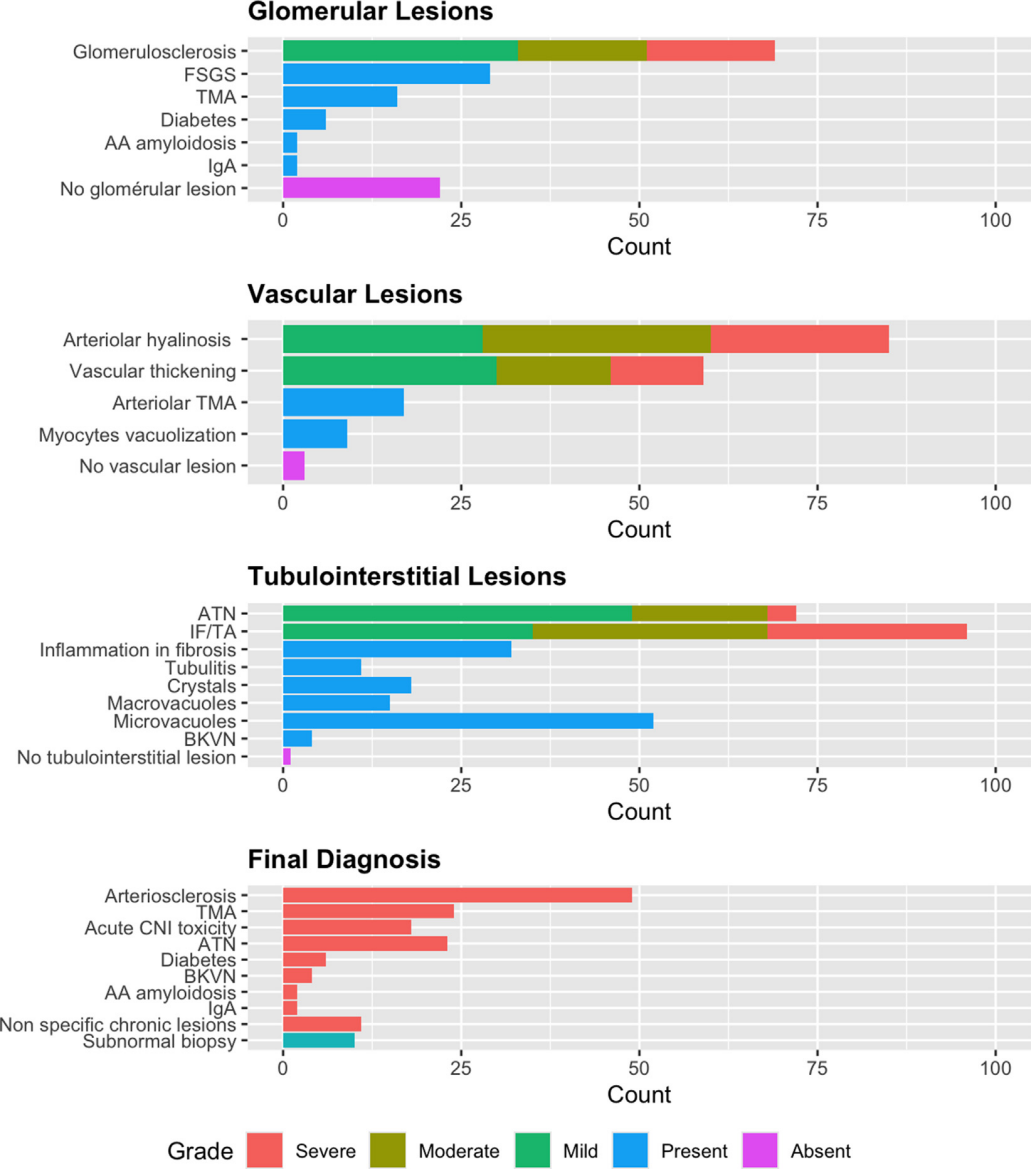
Histopathological findings on the kidney biopsy. See Supplementary Table S1 for the details of the scoring. AA, AA amyloidosis; ATN, acute tubular necrosis; BKVN, BK virus nephropathy; CNI, calcineurin inhibitors; FSGS, focal segmental glomerulosclerosis; IF/TA, interstitial fibrosis and tubular atrophy; TMA, thrombotic microangiopathy.

Renal biopsies revealed a wide spectrum of chronic lesions involving the 3 compartments: glomerular, vascular, and tubulointerstitial (Figure 2 and Supplementary Table S3).

### Glomerular Lesions

The mean percentage of glomerulosclerosis observed was 25.0% ± 25.8% (18.0% ± 23.7% after removal of maximal age-related glomerulosclerosis). Focal segmental glomerulosclerosis (FSGS) lesions were observed in 29% of patients, but no patients had clinical or histological features of primary FSGS. Six patients had lesions of diabetic glomerulosclerosis, and 2 had AA amyloidosis. Immunofluorescence staining was positive in only 2 patients with IgA. These IgA depositions were mainly mesangial, predominant compared to C3, and not associated with endocapillary proliferation, which does not suggest a postinfectious IgA nephropathy. Only 22 patients were devoid of significant glomerular lesions (glomerulosclerosis ≤5%, no specific lesion).

### Tubulointerstitial Lesions

All but 1 patient had significant tubulointerstitial lesions. ATN was the most frequent acute lesion observed in our cohort and it was present in 72% of patients.

Thirty-two patients displayed interstitial inflammation in fibrotic areas (inflammation in fibrous areas ≥1). For 11 of these patients, the inflammation involved more than 10% of the cortical area (total inflammation ≥1), and 2 patients also had significant inflammation in nonfibrotic areas (inflammation in nonfibrotic areas ≥1). Notably, none of these biopsies displayed eosinophils, which could suggest an immunoallergic mechanism.

Four patients had BK-virus nephropathy.

Ninety-six percent of patients had IF/TA, which was severe (>50% of the surface area) in 28% of patients.

Eighteen patients had intratubular and/or interstitial crystals, either nonrefractive crystals in polarized light suggestive of calcium phosphate (*n* = 12) or refractive crystals suggestive of calcium oxalate (*n* = 6). The number of crystals ranged from 0.13 to 1 per ×20 power field, with a median (interquartile range) value of 0.33 (0.2–0.7) corresponding to 1 crystal every three ×20 fields. The crystals were not associated with cystic fibrosis or ATN lesions.

We also noted the high prevalence of tubular vacuolization: 15 patients had tubular macrovacuolizations; and 52 patients had tubular isovolumetric vacuolization, including 18 with severe lesions that we considered suspicious of acute CNI toxicity.

### Vascular Lesions

Chronic vascular lesions were frequent and consisted of either ah and/or vascular fibrous intimal thickening. Only 4 biopsies were devoid of vascular lesions.

TMA was diagnosed in 24 biopsies, with at least an acute pattern (except for 2 patients), and consisted of exclusive glomerular TMA in 7 patients, exclusive arteriolar TMA in 8 patients and both arteriolar and glomerular TMA in 9 patients. Half of the patients with a histological TMA had biological signs of TMA, whereas among the 15 patients with a systemic TMA, 3 had no histological lesions.

### Final Histological Diagnosis

**Figure 3:**
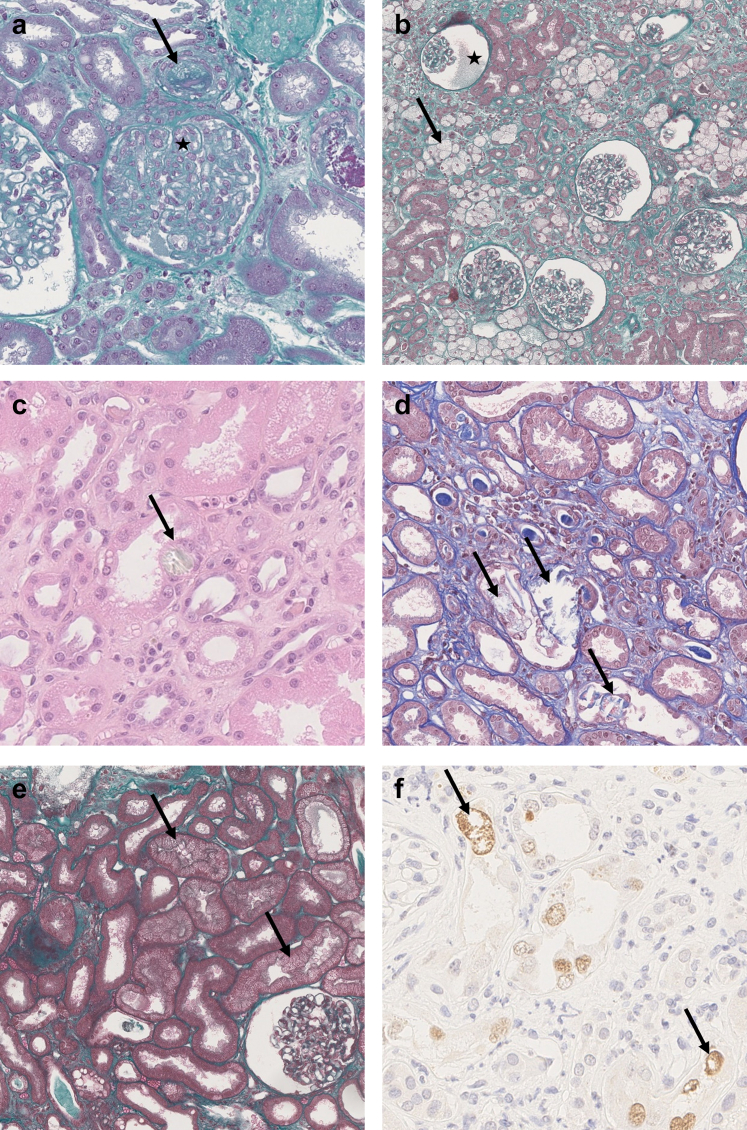
Acute kidney lesions. (a) Glomerular and arteriolar thrombotic microangiopathy with acute fibrin thrombi in the arteriolar lumen (arrow) and clear subendothelial spaces with double contours (star). Masson Trichrome, original magnification ×20. (b) Ischemic glomeruli with retracted flocculus in the Bowman space (star) and numerous tubular macrovacuolizations (arrow) in the cortex. Masson Trichrome, original magnification ×5. (c) Crystal (arrow) within the cytoplasm of an epithelial cell of a proximal tubule, HES, original magnification ×20. This crystal is refringent under polarized light. (d) None refringent crystals found in the tubular lumen of proximal tubules (arrows). Masson Trichrome, original magnification ×10. (e) Ischemic glomerulus and numerous tubular microvacuolizations in proximal tubules (arrows) suggestive of acute CNI toxicity. Masson Trichrome, original magnification ×10. (f) Immunohistochemistry with anti SV40 antibody showing several positive nuclei (arrows), in favor of a polyomavirus nephropathy. Original magnification ×40. CNI, calcineurin inhibitors.

**Figure 4:**
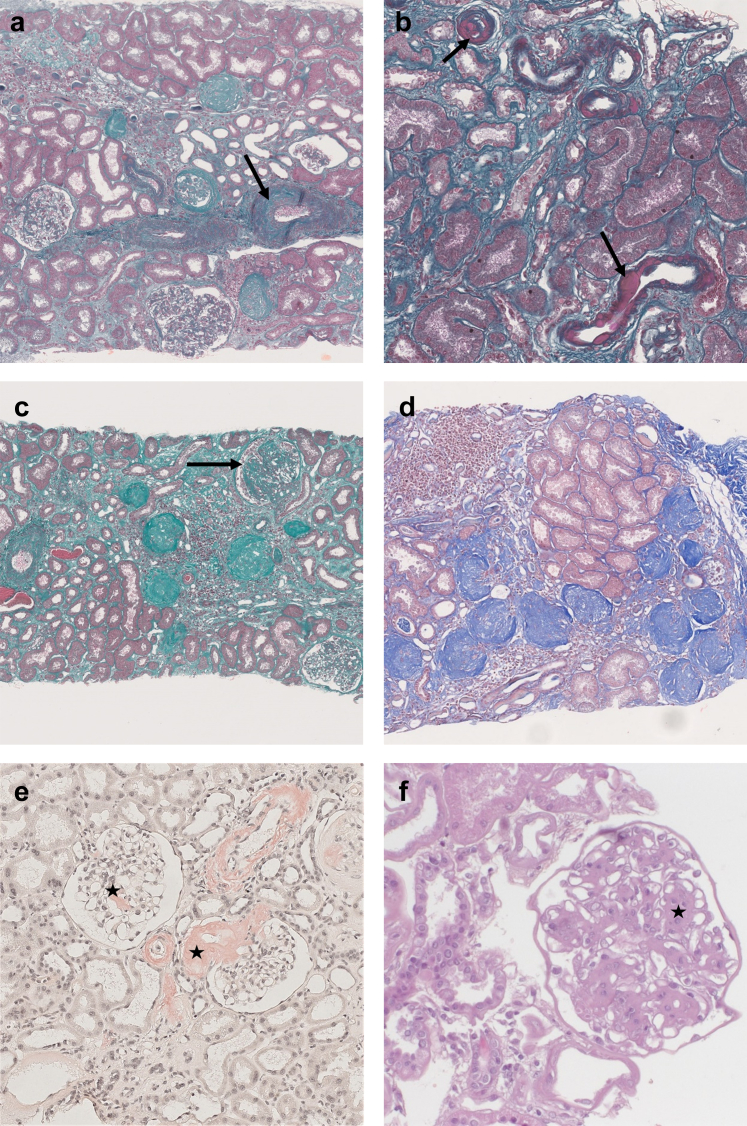
Chronic kidney lesions. (a) Interstitial fibrosis with severe arteriosclerosis (arrow). Masson Trichrome, original magnification ×5. (b) Severe multifocal arteriolar hyalinosis (arrows). Masson Trichrome, original magnification ×10. (c) Severe chronic lesions with several globally sclerotic glomeruli and a focal and segmental glomerulosclerosis (arrow). Masson Trichrome, original magnification ×5. Estimated GFR 96 ml/min per 1.73 m^2^, massive proteinuria 18.8 g/g. (d) Focal cortical atrophy in the subcapsular cortex containing numerous globally sclerotic glomeruli. Masson Trichrome, original magnification ×5. (e) Glomerular and vascular red Congo positive deposits in favor with the diagnosis of amyloidosis (stars). SAA protein immunohistochemistry was positive, in favor of AA amyloidosis. Congo Red staining, original magnification ×10. (f) Nodular glomerulosclerosis (star) in a diabetic nephropathy. HES, original magnification ×20.

In Figure 2, we show the final diagnosis that was blindly established by the renal pathologists, and in Figures 3 and 4, we present characteristic acute and chronic lesions, respectively. Forty-four patients had 2 or more final diagnoses. The most frequent were the following:

- For acute injuries, acute CNI toxicity and ATN (consisting of moderate to severe ATN);
- For chronic injuries, arteriosclerosis consisting of moderate to severe arterial intimal thickening; and
- TMA that may represent acute and/or chronic lesions.

Eleven patients had nonspecific chronic changes consisting of grade 2/3 IF/TA and/or grade 2/3 ah lesions without significant arteriosclerosis or any other final diagnosis. Eight of these patients had grade 2/3 ah lesions. Ten patients had a “subnormal” biopsy, displaying no or only mild elementary lesions.

### Clinicopathological Correlations

We next determined whether there were correlations between the histological lesions and several clinical features.

### Timing of Histological Lesion Observation

There was an association between the delay of KB after LT and the observed histological lesions (Supplementary Table S4). Chronic lesions (IF/TA, glomerulosclerosis, FSGS, and vascular lesions) were more prevalent and more severe in patients with late KB, performed more than 5 years after LT. Lesions indicative of acute injury (tubular isovolumetric vacuolization, and TMA) were more frequently observed in early biopsies within the first 2 years of LT; they were also observed in a significant proportion of late biopsies (38.7% of late biopsies displaying isometric vacuolization). Interestingly, ATN was detected in early and late biopsies.

### Renal Function and Proteinuria

**Figure 5:**
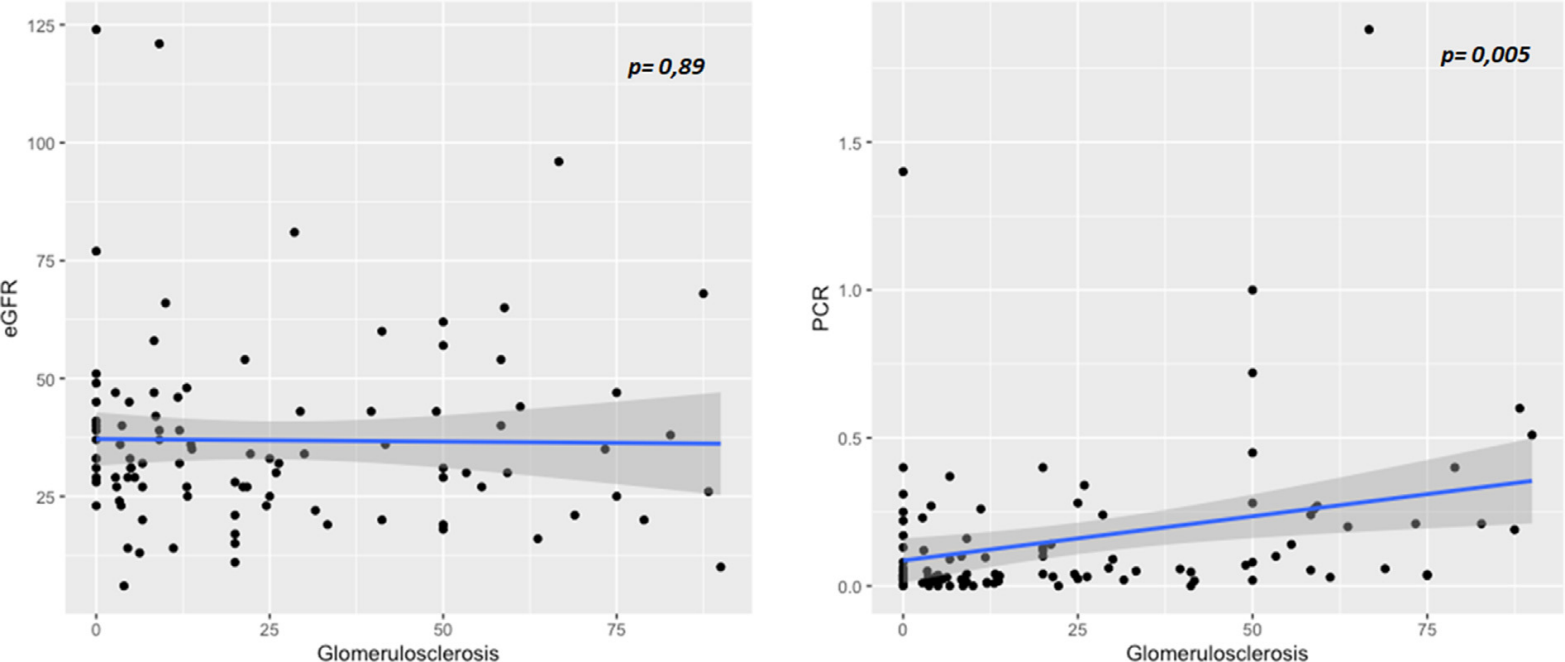
Correlation between estimated glomerular filtration rate (eGFR, ml/min per 1.73 m^2^) or PCR (protein-to-creatinine ratio, g/mmol) and percent of glomerulosclerosis (%). *P*-value corresponds to Pearson correlation test.

eGFR was only associated with moderate to severe IF/TA and TMA but not with other lesions (Supplementary Table S5); in particular, there was no correlation with the percentage of glomerulosclerosis (correlation, −0.01; *P* = 0.89, as illustrated in Figures 4c and 5). Proteinuria was associated with glomerulosclerosis (correlation, 0.28; *P* = 0.005), FSGS, and ATN (Figure 4 and Supplementary Table S5).

### Immunosuppressive Treatment and the Risk of TMA

Patients receiving an mTORi-CNI combination were at greater risk of histological TMA (odds ratio, 2.8 [1.1–7.2], *P* = 0.03) but not of systemic TMA, than patients receiving other immunosuppressive regimens (Supplementary Table S6). Moreover, the risk of developing systemic TMA was associated with higher tacrolimus trough levels at the time of KB (mean, 9.1 ± 3.1 ng/ml vs. 7.0 ± 2.8 ng/ml; *P* = 0.022). In contrast, patients treated with a standard CNI-mycophenolate combination had a greater incidence, but not significantly so, of acute CNI-induced lesions (odds ratio, 2.9 [0.9–9.5], *P* = 0.06).

### Other Correlations

Regarding the association of nephropathy with the indication for LT, diabetic nephropathy was observed only in patients with cystic fibrosis (all these patients were already treated for diabetes at the time of LT), and patients with pulmonary fibrosis were more frequently diagnosed with acute CNI toxicity lesions (35.0 vs. 13.7%, *P* = 0.027).

The 4 patients with BK virus nephropathy had positive blood BK viremia with a PCR ≥100.000 copies/ml at the time of diagnosis.

### Renal Outcome

Patients were followed-up with for 99.7 ± 64.8 months after LT and 50.5 ± 36.1 months after KB. Thirty-six patients (36%) died during the follow-up, including 2 who died early at 5- and 6-months post-LT from early postoperative complications.

Thirty-four patients (34%) in the cohort progressed to ESRD after a median time (interquartile range) of 76.2 (47.9–114.3) months since LT and 20.1 (8.9–29.9) months since KB. KBs were performed later after LT in patients who experienced ESRD: 69.6 ± 60.1 versus 39.9 ± 44.4 months (*P* = 0.006). Twelve of them (35.3%) died during follow-up.

**Figure 6:**
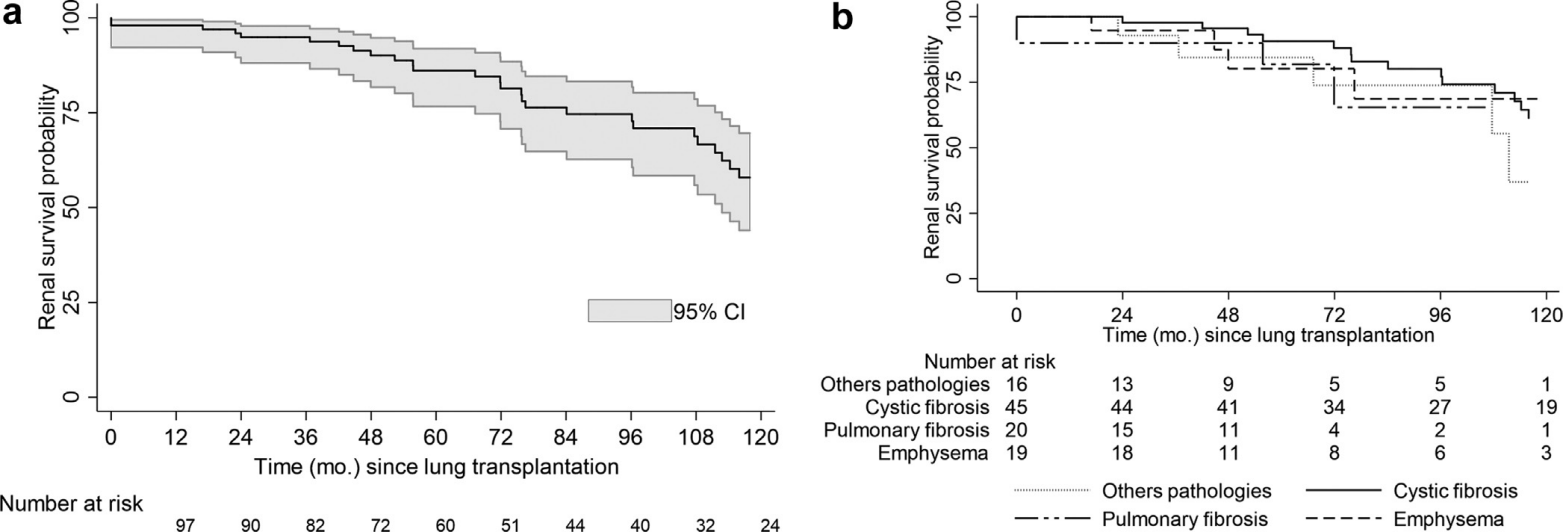
(a) Kaplan Meier estimate of renal survival after lung transplantation (months). (b) Kaplan Meier estimate of renal survival after lung transplantation by pulmonary diseases. Survivals were compared with the log-rank test (*P* = NS).

The probability of renal survival at 10 years post-LT was 57.9% (95% confidence interval: 46.3–72.4) (Figure 6a). The time between the occurrence of the first biological sign of renal injury and ESRD was 62.7 months, which is indicative of a rapid decline in kidney function. Pulmonary disease was not associated with renal survival after LT (log rank test, *P* = 0.59, Figure 6b). Among the 42 patients who were alive and not with ESRD, the mean eGFR at the last follow-up was 44.4 ± 21.6 ml/min per 1.73 m^2^. Fifteen patients underwent renal transplantation during the follow-up.

Among the 24 patients with a final diagnosis of TMA, 10 patients had no change, and 14 patients had a decrease in CNI (*n* = 8) and/or discontinued or minimized mTORi (*n* = 9). Regarding their evolution, 12 of the 14 patients with a minimization of immunosuppression had an improvement in their eGFR (1 patient died, and 1 had a stable eGFR); whereas among the 10 patients with unchanged treatment, only 3 had an improvement in their eGFR, 2 had a stabilization, 4 had a degradation (2 reaching ESRD), and 1 died.

Seventy-six patients (76%) were treated for an episode of acute or chronic rejection, with or without histological confirmation. Compared to patients without rejection, there was no difference in renal survival or in the mean eGFR at the last follow-up.

We analyzed the associations of clinical and histological lesions with renal survival in patients with KB. In Supplementary Table S7, we display the results of the univariate Cox model. The clinical parameters associated with renal failure were postoperative hemodialysis, diabetic status at KB, a PCR >3 g/g, the time from signs of renal injury to KB and the time from LT to KB. The histological lesions associated with renal failure were the percentage of glomerulosclerosis, the presence of FSGS lesions, and a diagnosis of diabetic nephropathy. According to the final multivariate Cox model (Table 2), after adjustment, the only histological lesion associated with the risk of ESRD was a percentage of glomerulosclerosis >4%. The 2 clinical variables independently associated with ESRD were postoperative dialysis and the PCR.

**Table 2:** Factors associated with ESRD in the Cox multivariable analysis

Factors	Number of patients	Number of events	HR	95% CI	*P*-value
Percentage glomerulosclerosis					
(0–4.409)	22	1			
(4.409–13.4)	22	5	15.5	1.42–169	0.024
(13.4–43.5)	22	8	24.0	2.32–248	0.008
(43.5–90)	18	11	245	3.41–311	0.002
Postoperative dialysis					
No	74	20			
Yes	10	5	3.45	1.04–11.5	0.044
Urinary protein/creatinine ratio (g/mmol)					
(0–0.3)	73	17			
(0.3–1.88)	11	8	14.1	3.36–59.0	<0.001

ATN, acute tubular necrosis; CI, confidence interval; eGFR, estimated glomerular filtration rate by chronic kidney disease-epidemiology collaboration 2021; ESRD, end-stage renal disease; HR, hazard ratio; KB, kidney biopsy; TMA, thrombotic microangiopathy.

Eighty-four patients had no missing data for all the parameters in the Cox model. The final multivariate Cox model was obtained by entering risk factors from the univariate model (Supplementary Table S6) that achieved *P* ≤ 0.10 as the thresholds in a single multivariate proportional hazards model. The final multivariate model was adjusted for the following parameters: focal and segmental glomerulosclerosis, interstitial fibrosis and tubular atrophy, inflammation in fibrous sections, arteriolar hyalinosis, acute tubular necrosis, diabetes at KB, eGFR at KB, systemic signs of TMA.

## Discussion

The large number of biopsies analyzed in our present study allowed us to reveal the very large spectrum of lesions observed in LT recipients, involving all compartments. We made the following remarkable observations:

- The high frequency and severity of chronic vascular and tubulointerstitial lesions, even in the absence of hypertension or diabetes.
- The unexpected frequency of some specific lesions such as BK virus nephropathy, calcic crystals, and TMA, whose occurrence was associated with the combination of mTORi/CNI; and
- The observation of acute lesions (TMA, ATN, and microvacuolizations) in late biopsies was performed after 5 years.

In comparison to 2 previous studies on the renal histology of LT recipients,^21^^,^^22^ our study highlights several new findings. The comparison of histological lesions between studies is challenging due to the lack of standardization, and the strength of our study is the large number of biopsies studied by 3 pathologists with a common reading grid based on a semiquantitative scoring system inspired by the latest Banff classification of kidney transplant histological lesions.^26^ The association of the original pulmonary pathology with renal lesions has never been investigated; the only study to report this information included only cystic fibrosis transplant patients.^21^Analyzing biopsies performed in 4 large transplant centers allowed us to include a wide range of patients and lung diseases. In our cohort, we did not find a relationship between lung pathology and the main renal histological diagnosis, except that all patients with diabetic nephropathy had cystic fibrosis.

We focused on several renal injuries, such as CNI nephrotoxicity, TMA and BK-virus nephropathy because early diagnosis and intervention in these patients may slow the progression of renal dysfunction.

“Acute CNI nephrotoxicity” was the main diagnosis in 18 patients and consisted of the presence of tubular isometric vacuolization. Interestingly, these lesions have also been observed in late biopsies and tubular vacuolization may be observed in other clinical settings, such as renal ischemia or intravenous administration of hyperosmotic fluids or immunoglobulins, although in this latter case, “osmotic nephrosis” is distinguished by our pathologists by the varying size of the vacuoles.

Our pathologists did not assess chronic CNI-related nephrotoxicity because of the lack of day 0 biopsies (before CNI exposure) and the lack of specific histological signs. Eight patients with nonspecific chronic lesions had arteriolar hyalinosis lesions without arteriosclerosis, which may correspond to chronic CNI nephrotoxicity. However, we have shown in a previous study on kidney transplant recipients that even patients who were not treated with CNI had lesions that were blindly established as CNI nephrotoxicity.^27^ In particular, arteriolar hyalinosis can be observed in patients with hypertension and/or diabetes, and the nodular pattern is not a reliable sign.^28^ Considering that 61% of patients had hypertension and 51% had diabetes at the time of KB, we preferred to report the frequency of arteriolar lesions without determining their toxic origin. Interestingly, if we only consider moderate to severe ah, the frequency (57%) is close to that observed (61%) in the 30 LT recipients in the study by Schwarz *et al.*^22^ Even if the mechanism of these tubular and vascular lesions is not univocal, identifying these long-term ongoing lesions, particularly chronic vascular lesions, is of paramount importance for determining the optimal dosage of CNI and limiting its toxicity.

We confirmed the frequent incidence of TMA in this population (24%), which has already been described as affecting 14.0%^22^ and 46.5%^21^of patients in the 2 other studies cited above. We also observed that histological lesions were associated with biological signs of TMA in only half of the patients and that the risk of TMA almost tripled when using the CNI-mTORi combination. TMA has indeed been reported in patients receiving mTORi alone or in combination with CNIs, with the risk being markedly greater in the latter group. Many reports have shown resolution after mTORi discontinuation.^29^^,^^30^ The most important mechanism is likely the mTORi-induced downregulation of vascular endothelial growth factor, which may directly cause TMA or impair the repair of endothelial damage caused by CNIs.^31^ No cases of TMA were reported in the first large trials investigating the use of a combination of CNI, everolimus, mycophenolate, and steroids for nephroprotection in LT recipients.^15^ However, CNI concentrations are probably not sufficiently lower after LT than after kidney transplantation, which may explain the greater incidence of TMA in patients receiving this combination.

Overall, the incidence of BK virus nephropathy was 4% in our cohort, leading to ESRD in three-quarters of patients, which is higher than the 1.3% reported recently.^31^ We observed systematically positive serum replication in patients which supports the screening of BK virus in all LT recipients.

We observed crystals in 18 patients (either calcium oxalate or calcium phosphate), whereas in 15 patients with cystic fibrosis, Lefaucheur *et al.*^21^ observed crystals in 60% and concluded that oxalic nephropathy occurred in 33%. We did not use this terminology because of the lack of a clear definition, and importantly, the presence of these crystals was not correlated with cystic fibrosis in our study.

Several observations suggest that patients should be referred early to nephrologists in the case of signs of kidney disease (proteinuria, lack of complete AKI recovery, and decrease in eGFR) to discuss KB. Indeed, in our study, the time lag (median 15 months) between the appearance of biological signs of renal disease (renal insufficiency and/or proteinuria) and the biopsy performed showed a certain inertia. This delay may have contributed in some cases to the severity of the chronic lesions observed. Moreover, eGFR may be falsely reassuring for many patients. It did not correlate with glomerulosclerosis as expected. This finding supports the study by Florens *et al.*,^32^ which showed that the eGFR overestimates renal function with a bias of 18 ml/min per 1.73 m^2^ compared to the measured GFR before LT. Measurements of the GFR before LT and 1 year after revealed a dramatic decrease of 48 ml/min per 1.73 m^2^. The pretransplant measured GFR had better predictive value than the eGFR for predicting CKD stage 3 at 1 year.

Moreover, some lesions may necessitate therapeutic modifications such as CNI minimization in the case of arterial or arteriolar lesions, a decrease in global immunosuppression in the case of BK virus nephropathy, and discontinuation of mTORi in the case of TMA. The impact of these therapeutic changes cannot be analyzed in our study because of its retrospective nature, which does not allow us to measure the real impact of these changes.

Eventually, referral to a nephrologist is important to start nephroprotective treatment (strict blood pressure control in the case of hypertension, optimal diabetic balance, and antiproteinuric treatment if necessary). At the time of KB, half of the patients had a PCR greater than 0.5 g/g, and half of the patients had an eGFR ≤30 ml/min per 1.73m^2^. With such renal markers, KDIGO guidelines recommend for patients with CKD, monitoring of proteinuria and eGFR at least 3 times per year.^33^ The use of new therapeutics such as gliflozins has not been studied in this population but could be very promising,^34^ particularly if they are introduced early in the course of renal disease.^35^

In conclusion, this large renal pathological study revealed a great diversity of renal lesions after LT. We can conclude from our study that the kidney lesions that may arise following LT are very heterogeneous and often unpredictable in their nature and severity. These patients should be referred to a nephrologist in a timely manner in the event of renal disease, to discuss the possibility of KB and the initiation of early nephroprotective measures.

## Disclosure

All the authors declared no competing interests.

## Supplementary Materials

Supplementary File (PDF) **Table S1.** Definition of elementary histological lesions and main diagnosis. **Table S2.** Characteristics of the population at the time of lung transplantation. **Table S3.** Histopathological finding on the kidney biopsy. **Table S4.** Association between the delay of lung transplantation/kidney biopsy and clinical/histological parameters at kidney biopsy. **Table S5.** Association between eGFR or PCR and histological parameters at kidney biopsy. **Table S6.** Association between the immunosuppressive treatment and clinical/histological parameters at kidney biopsy. **Table S7.** Univariate Cox model for kidney failure since the kidney biopsy. **STROBE Statement.**

## Footnotes

Supplementary File (PDF)
**Table S1.** Definition of elementary histological lesions and main diagnosis. **Table S2.** Characteristics of the population at the time of lung transplantation. **Table S3.** Histopathological finding on the kidney biopsy. **Table S4.** Association between the delay of lung transplantation/kidney biopsy and clinical/histological parameters at kidney biopsy. **Table S5.** Association between eGFR or PCR and histological parameters at kidney biopsy. **Table S6.** Association between the immunosuppressive treatment and clinical/histological parameters at kidney biopsy. **Table S7.** Univariate Cox model for kidney failure since the kidney biopsy.
**STROBE Statement.**
