# Supplementary material for: Kidney Biopsy Findings After Lung Transplantation
Source: Kidney Int Rep. 2024 Jul 6;9(9):2774–85. doi: 10.1016/j.ekir.2024.07.005 (PMC11403037; doi:10.1016/j.ekir.2024.07.005)
Supplement: Supplementary File (PDF) — Table S1. Definition of elementary histological lesions and main diagnosis. Table S2. Characteristics of the population at the time of lung transplantation. Table S3. Histopathological finding on the kidney biopsy. Table S4. Association between the delay of lung transplantation/kidney biopsy and clinical/histological parameters at kidney biopsy. Table S5. Association between eGFR or PCR and histological parameters at kidney biopsy. Table S6. Association between the immunosuppressive treatment and clinical/histological parameters at kidney biopsy. Table S7. Univariate Cox model for kidney failure since the kidney biopsy. STROBE Statement. [file mmc1.pdf]

Supplementary Table S1: Definition of elementary histological lesions and main diagnosis

Elementary lesions definition	
**Glomeruli**	
**Globally sclerotic glomeruli**	Sclerotic remnant of glomerulus consisting of extracellular material.
**Ischemic glomeruli**	Glomerulus showing collapse of capillary tuft ± thickening of Bowman's capsule.
**Glomerular thrombotic microangiopathy**	Includes acute features: fibrinoid necrosis, mesangiolysis, fibrinoid thrombi in the glomerular capillaries, endothelial swelling, and chronic features: duplication of the glomerular basement membrane.
**Focal and segmental glomerulosclerosis**	Presence of segmental scarring that involves a part of the glomerulus.
**Vessels**	
**Arteriolar hyalinosis**	Accumulation of eosinophilic, amorphous material in arteriolar wall. The extent of arteriolar hyalinosis is evaluated according to the Banff ah lesion:<br>**ah0**—No arteriolar hyalinosis<br>**ah1**—Hyalinosis in only 1 arteriole, without circumferential involvement.<br>**ah2**—Hyalinosis in more than 1 arteriole, without circumferential involvement.<br>**ah3**—Hyalinosis with circumferential involvement, independent of the number of arterioles involved.
**Arteriolar thrombotic microangiopathy**	Includes acute features: fibrin thrombi, intimal mucoid edema, and chronic features: intimal fibrosis with fibrous arteriolar occlusion.
**Arteriolar myocyte vacuolization**	Vacuolization of the smooth muscle cells of the media of arterioles and small arteries.
**Arteriosclerosis**	Accumulation of fibrous tissue in the intima resulting in intimal thickening. May be associated with intimal fibroelastosis. The extent of arterial intimal fibrosis is evaluated according to the Banff cv lesion:<br>**cv0**—No chronic vascular changes.<br>**cv1**—Vascular narrowing of up to 25% luminal area by fibrointimal thickening.<br>**cv2**—Vascular narrowing of 26 to 50% luminal area by fibrointimal thickening.<br>**cv3**—Vascular narrowing of more than 50% luminal area by fibrointimal thickening.
**Fibrous arterial occlusion**	Occlusion of the lumen of arteries may be related to severe intimal thickening or to occlusive thrombosis.
**Arterial thrombosis**	Includes recent or chronic recanalized thrombosis in arteries.
**Mucoid intimal thickening**	Accumulation of edematous extracellular matrix in intima.
**Tubulointerstitial compartment**	
**Interstitial fibrosis and tubular atrophy (IF/TA)**	Expansion of normal interstitial connective tissue by increased collagen, usually accompanied by tubular atrophy. IF/TA is assessed as a percentage of the cortical area<br>**IF/TA 1**: 6% ≤ci <25% and/or 0%<ct<25%<br>**IF/TA 2**: 25 ≤ci<50% and/or 25≤ct<50%<br>**IF/TA 3**: 50≤ci and/or 50%≤ct
**Inflammation (i)**	Degree of inflammation in nonscarred areas of cortex.<br>**i0**—No inflammation or less than 10% of unscarred cortical parenchyma<br>**i1**—Inflammation in 10% to 25% of unscarred cortical parenchyma<br>**i2**—Inflammation in 26% to 50% of unscarred cortical parenchyma<br>**i3**—Inflammation in >50% of unscarred cortical parenchyma
**Total inflammation (ti)**	Evaluates the extent of total cortical inflammation in scarred and unscarred cortical parenchyma
**Inflammation in fibrosis (i-IFTA)**	Evaluates the extent of inflammation in scarred cortex<br>**i-IFTA0**—No inflammation or less than 10% of cortical parenchyma with interstitial fibrosis and tubular atrophy.<br>**i-IFTA1**—Inflammation in 10% to 25% of cortical parenchyma with interstitial fibrosis and tubular atrophy.

	**i-IFTA2**—Inflammation in 26% to 50% of cortical parenchyma with interstitial fibrosis and tubular atrophy.<br>**i-IFTA3**—Inflammation in >50% of cortical parenchyma with interstitial fibrosis and tubular atrophy.
**Tubulitis (t)**	Presence of mononuclear cells in the basolateral aspect of the renal tubule epithelium. Lesion score t should only be scored in "preserved" areas of cortex, defined as areas without interstitial fibrosis, in non-atrophic, mildly atrophic and moderately atrophic tubules.<br>**t0**—No mononuclear cells in tubules or single focus of tubulitis only.<br>**t1**— 2 or more foci with 1 to 4 mononuclear cells/tubular cross section (or 10 tubular cells).<br>**t2**—2 or more foci with 5 to 10 mononuclear cells/tubular cross section (or 10 tubular cells).<br>**t3**—2 or more foci with >10 mononuclear cells/tubular cross section or the presence of ≥2 areas of tubular basement membrane destruction accompanied by i2/i3 inflammation and t2 elsewhere.
**Pvl (polyoma virus load)**	Evaluates the percentage of tubules in the biopsy with at least one viral inclusion and/or positive staining for SV40 antigen.<br>**pvl 1**: <1% of the tubules<br>**pvl 2**:1-10% of the tubules<br>**pvl 3**: >10% of the tubules
**Crystals**	Crystals were observed with polarization and classified as refringent or non-refringent crystals. The number of crystals per x20 power field was given and corresponded to the ratio of the total number of crystals observed on the number of x20 power fields in the biopsy.
**Macrovacuolizations**	Macrovacuolizations were defined as voluminous vacuoles, in swollen tubular cells with picnotic nuclei.The extent of macrovacuolization was scored according to the fraction of tubules with vacuoles at x10 magnification<br>0: absent<br>1: <25%<br>2: 25-50%<br>3: >50%
**Microvacuolizations**	Microvacuolizations were defined as small isometric vacuoles. The extent of microvacuolization was scored according to the fraction of tubules with vacuoles at x10 magnification<br>0: absent<br>1: <25%<br>2: 25-50%<br>3: >50%
**Acute tubular necrosis (ATN)**	ATN was defined as tubular luminal dilatation, simplification of the lining epithelium and loss of the brush border in proximal tubules with sometimes denudation of the basement membrane. ATN extent was scored according to the percentage of cortical surface with lesions:<br>0: absent<br>1: <25%<br>2: 25-50%<br>3: >50%

Definition of main diagnosis

Diabetes: diagnosed as the presence of nodular PAS positive Jones positive glomerulosclerosis with negative immunofluorescence
IgA nephropathy: diagnosed in the presence of predominant IgA mesangial deposits by immunofluorescence
Thrombotic microangiopathy: diagnosed in the presence of glomerular and/or arteriolar TMA lesions
Amyloidosis: diagnosed in the presence of Congo red positive deposits
BK virus nephropathy: diagnosed in the presence of BK viral inclusions and/or SV40 positive staining
Arteriosclerosis: defined as the presence of cv lesions of 2 or 3
Acute tubular necrosis: defined as the presence of ATN lesions of 2 or 3
Acute CNI toxicity: was arbitrary defined as the presence of microvacuolizations lesions of 2 or 3

Crystalline nephropathy: defined as the presence of at least one crystal
No specific chronic lesions: defined as the presence of isolated grade 2/3 IFTA and/or grade 2/3 ah lesions without significant arteriosclerosis (cv0 or 1) or other diagnosis
Subnormal kidney: biopsy not fulfilling the above criteria

Supplementary Table S2: Characteristics of the population at the time of lung transplantation

Before surgery		Patients (n=100)	MD
Sex (females), n (%)		45 (45)	0
Location of transplantation, n (%)			0
	Center 1	33 (33)	
	Center 2	35 (35)	
	Center 3	22 (22)	
	Center 4	10 (10)	
Year of transplantation, n (%)			0
	Before 2000	2 (2)	
	2000-2004	7 (7)	
	2005-2009	19 (19)	
	2010-2014	31 (31)	
	2015-2019	36 (36)	
	2020-2021	5 (5)	
Pathologies leading to transplantation, n (%)			0
	Cystic fibrosis	45 (45)	
	Pulmonary fibrosis	20 (20)	
	Emphysema after tobacco exposure	19 (19)	
	Other*	16 (16)	
Age (years), median [IQR] (min-max)		40.4 [25.8-54.5] (13-66)	0
BMI, median [IQR]		20.3 [18.0-23.0]	14
Diabetes mellitus, n (%)		19 (19.6)	3
Arterial hypertension, n (%)		13 (14.3)	9
eGFR (ml/min/1.73m²), median [IQR]		112 [98.5-129]	8
Presence of donor specific antibody (MFI≥500), n (%)		34 (57.6)	41
**Surgery**			
Super-emergency procedure, n (%)		16 (16)	0
Bi-pulmonary graft, n (%)		91 (91)	0
Max. graft ischemia time (mins), mean (SD)		380.9 (125)	7
Extra-corporeal circulation, n (%)			9
	CPB	28 (30.8)	
	ECMO**	34 (37.4)	
	None	30 (32.9)	
**Post-surgery**			
Extubation before 72 hours, n (%)		46 (48.9)	7
Acute Kidney Injury, n (%)			12
	No	17 (19.3)	
	KDIGO 1	31 (35.2)	
	KDIGO 2	13 (14.8)	
	KDIGO 3 (Including hemodialysis)	27 (30.7)	
	Hemodialysis, n (%)	13 (13.7)	5
**Immunosuppressive treatment**			
Induction therapy, n (%)		52 (56.5)	8
	rATG	36 (69.0)	
	Basiliximab	16 (31.0)	
Maintenance treatment, n (%)			3
	Steroids	97 (100)	
	Tacrolimus	80 (82.5)	
	Cyclosporine	17 (17.5)	
	Mycophenolate	88 (90.7)	
	Azathioprine	8 (8.3)	
	M-Tor inhibitors	2 (2.1)	

MD: Missing data. * Alpha1 antitrypsin deficit (n=3), Pulmonary arterial hypertension (n=5), bronchiectasis (n=3), bronchiolitis (n=3), hemosiderosis (n=1), pleuroparenchymal fibroelastosis (n=1). IQR: Interquartile range. BMI: Body mass index. eGFR:

Estimated Glomerular rate filtration by CKD EPI 2021 or SCHWARTZ formula. MFI: Mean fluorescence intensity. rATG: Rabbit anti thymocyte globulin. CBP: Cardiopulmonary bypass, **ECMO: Extra corporal membrane oxygenation (initiated on pre-operative for transplantation in super-emergency for 6 patients).

Supplementary Table S3: Histopathological finding on the kidney biopsy

Glomerular involvement		n	MD
**% of glomerulosclerosis**	≤5	31	0
	5-25	33	
	25-50	18	
	> 50	18	
**Focal segmental glomerulosclerosis**		29	0
**Diabetes**		6	1
**AA Amyloidosis**		2	0
**Glomerular ischemia (>1)**		66	0
**Glomerular TMA**		16	0
	Acute	15	
	Acute and chronic	1	
**Immunofluorescence IgA staining**		2	7

Vascular involvement		n	MD
**Arteriolar hyalinosis**		85	2
	Mild	28	
	Moderate	32	
	Severe	25	
**Vascular fibrous intimal thickening**		79	8
	Mild (narrowing <= 25%)	30	
	Moderate (26 and 50%)	36	
	Severe (narrowing > 50%)	13	
**Combined moderate to severe arteriolar and arterial lesions**		36	9
**Arteriolar TMA**		17	0
	Acute	10	
	Chronic	3	
	Acute and chronic	4	
**Myocytes vacuolization**		9	1

Tubulointerstitial involvement		n	MD
**Acute Tubular Necrosis**		72	2
	Mild	49	
	Moderate to severe	23	
**IF/TA**		96	0
	Mild (5-25% of the cortical surface)	35	
	Moderate (25-50%)	33	
	Severe (>50% of the cortical surface)	28	
**Inflammation in fibrous areas > 10%**		32	0
**- Associated with Inflammation in non-fibrous areas > 10%**		2	
**- Total inflammation representing >10% of cortical area**		11	
**BK virus nephropathy**		4	0
**Tubulitis**		11	0
**Crystals**		18	0
	Calcium oxalate	6	
	Calcium phosphate	12	
**Tubular macrovacuolization**		15	2
**Tubular isovolumetric vacuolization**		52	2
	Mild	34	
	Moderate to severe	18	

Final diagnoses	n
**Arteriosclerosis**	49
**TMA**	24
**Acute CNI toxicity**	18
**Acute tubular necrosis**	23
**Diabetes**	6
**BK-virus nephropathy**	4
**AA amyloidosis**	2
**IgA nephropathy**	2
**Nonspecific chronic lesions**	11
**“Subnormal” biopsy**	10

MD: Missing data, TMA: Thrombotic microangiopathy, IF/TA: Interstitial fibrosis Tubular atrophy, CNI: Calcineurin Inhibitors

Supplementary Table S4: Association between the delay of lung transplantation/kidney biopsy and clinical/histological parameters at kidney biopsy

	**Early kidney biopsy (< 2 years)**	**Middle-term kidney biopsy (2 – 5 years)**	**Late kidney biopsy (> 5 years)**	P value
n	46	22	32	
% of glomerulosclerosis, median [IQR]	**5 [0-13]**	10 [5-25]	**52.8 [41-68]**	**p<0.001**
FSGS, n (%)	6 (13)	4 (18.2)	**19 (59.4)**	**p<0.0001**
Histologic TMA, n (%)	**16 (34.8)**	4 (18.2)	4 (12.5)	**p=0.020**
Moderate to severe IF/TA, n (%)	**22 (47.3)**	15 (68.2)	24 (75)	**p=0.013**
IF/TA score, mean (SD)	**1.6 (0.9)**	2.0 (0.8)	2.1 (0.8)	**P=0.01**
Moderate to severe CV, n (%)	21 (45.7)	8 (36.4)	20 (62.5)	NS
CV score, mean (SD)	1.52 (0.9)	1.25 (1.0)	1.73 (0.8)	NS
Moderate to severe AH, n (%)	22 (47.8)	10 (45.4)	**25 (78)**	**p=0.003**
AH score, mean (SD)	1.5 (1)	1.3 (0.8)	**2.3 (0.9)**	**P<0.001**
Moderate to severe ATN, n (%)	12 (26.1)	6 (27.3)	5 (15.6)	NS
ATN score, mean (SD)	1 (0.8)	1.1 (0.7)	0.8 (0.7)	NS
Tubular microvacuolization, n (%)	**30 (66.7)**	10 (45.5)	12 (38.7)	**P=0.013**
Tubular macrovacuolization, n (%)	4 (8.9)	4 (18.2)	7 (22.6)	NS
Myocyte vacuolization, n (%)	7 (15.2)	0	2 (6.45)	NS
Presence of crystals, n (%)	10 (21.8)	4 (18.2)	4(12.5)	NS
Acute CNI toxicity, n (%)	11 (23.9)	4 (18.2)	3 (9.4)	NS
BK virus nephropathy, n (%)	1 (2.2)	2 (9.1)	1 (3.1)	NS
Diabetic nephropathy, n (%)	1 (2.2)	1 (4.6)	4 (12.9)	NS
eGFR (ml/min/1.73 m^2), mean (SD)	34.1 (17.5)	36 (23.9)	41.3 (20.1)	NS
Therapeutic impact of KB, n (%)	26 (56.5)	11 (50)	**10 (31.3)**	**p=0.03**

*** p value for Chi2/T student for categorial value or Wilcoxon/ttest for continues values.** IQR: Interquartile range. SD: Standard deviation. FSGS: Focal segmental glomeruosclerosis. TMA: Thrombotic microangiopathy IF/TA: Interstitial fibrosis tubular atrophy. CV: vascular fibrous intimal thickening. AH: Arteriolar hyalinosis. ATN: Acute tubular necrosis. CNI: Calcineurin inhibitors. BKV: BK virus. eGFR: Estimated Glomerular filtration rate by CDK EPI 2021. KB: Kidney biopsy

Supplementary Table S5: Association between eGFR or PCR and histological parameters at kidney biopsy

	**eGFR at the KB (ml/min/1.73m²), mean (SD)**			**PCR (g/g), median [IQR]**		
Histological Parameters	***Absent***	***Present***	***pValue****	***Absent***	***Present***	***pValue*****
FSGS	36.5 (19.7)	37.7 (21.0)	p=0.80	0.38 [0.2-1.2]	2.1 [0.8-3.7]	**p<0.001**
Moderate to severe IF/TA	43.8 (24.7)	32.7 (15.3)	**p=0.007**	0.6 [0.2-1.30]	0.55 [0.3-2.6]	p=0.56
Moderate to severe AH	38.2 (20.1)	35.9 (20.1)	p=0.58	0.6 [0.2-2.1]	0.6 [0.2-2.4]	p=0.88
Moderate to severe CV	39.5 (23.0)	34.0 (15.8)	p=0.17	0.5 [0.2-1.6]	0.7 [0.3-2.5]	p=0.18
Moderate to severe ATN	36.8 (16.8)	37.1 (29.0)	p=0.94	0.5 [0.2-1.8]	1.0 [0.4-4.0]	**p=0.026**
Histological TMA	39.2 (20.1)	29.6 (18.4)	**p=0.04**	0.5 [0.2-2.0]	1.0 [0.4-2.7]	p=0.11
Presence of crystals	37.7 (21.1)	33.3 (14.4)	p=0.41	0.5 [0.2-2.3]	0.6 [0.2-1.2]	p=0.87

*** p value for t-tests. ** p value for Wilcoxon test**. eGFR: Estimated Glomerular filtration rate by CKD EPI 2021. PCR: urinary protein/creatinine ratio. SD: Standard deviation. IQR: Interquartile range. IF/TA: Interstitial fibrosis tubular atrophy. AH: Arteriolar hyalinosis. CV: vascular fibrous intimal thickening. ATN: Acute tubular necrosis. TMA: Thrombotic microangiopathy

Supplementary Table S6: Association between the immunosuppressive treatment and clinical/histological parameters at kidney biopsy

KB traitements	CNI-MMF/AZA-STEROIDS	CNI-mTORi +/- MMF/AZA +/- STEROIDS	OTHERS	P value
Patients, n	59	32	9	
eGFR (ml/min/1.73 m²), mean (SD)	36.4 (20.4)	39.7 (21.6)	29.8 (5.8)	NS
PCR (g/g), median [IQR]	0.5 [0.2-1.8]	1.0 [0.4-2.7]	0.6 [0.2-1.4]	NS
Systemic TMA, n (%)	8 (13.6)	7 (21.9)	0	NS
Glomerular TMA, n (%)	8 (13.6)	6 (18.8)	2 (22.2)	NS
Arteriolar TMA, n (%)	7 (11.9)	**9 (28.1)**	1 (11.1)	**P=0.042**
Total histologic TMA, n (%)	10 (17.0)	**12 (37.5)**	1 (22.2)	**P=0.03**
Acute CNI toxicity, n (%)	14 (23.7)	3(9.4)	1(11.1)	NS
Glomerulosclerosis percent, mean (SD)	21.5 (26.1)	26.9 (23.3)	40.7 (28.6)	NS
FSGS, n (%)	14 (23.7)	13 (40.6)	2 (22.2)	NS
Moderate to severe IF/TA, n (%)	35 (59.3)	20 (62.5)	6 (66.7)	NS
Moderate-severe CV, n (%)	31 (52.5)	15 (46.9)	3 (33.3)	NS
Moderate-severe AH, n (%)	34 (57.6)	19 (59.4)	4 (44.4)	NS
Moderate-severe ATN, n (%)	17 (28.8)	5 (15.6)	1 (11.1)	NS
Presence of crystals, n (%)	11 (18.6)	5 (15.6)	2 (22.2)	NS
Time between LT and KB (months), mean (SD)	**41.3 (6.6)**	57.2 (8.0)	81.2 (22.8)	**P=0.045**

*** p value for Chi2/T student for categorial value or Wilcoxon/ttest for continues values.** eGFR: Estimated Glomerular filtration rate by CDK EPI 2021. KB: Kidney biopsy. CNI: Calcineurin inhibitor. mTORi: mTOR inhibitors. SD: Standard deviation. IQR: Interquartile range. PCR: urinary protein/creatinine ratio. IQR: Interquartile range. TMA: Thrombotic microangiopathy. FSGS: Focal segmental glomeruosclerosis. IF/TA: Interstitial fibrosis tubular atrophy. AH: Arteriolar hyalinosis. CV: vascular fibrous intimal thickening. ATN: Acute tubular necrosis. LT: Lung transplantation.

**Details of treatments for patients identified as “Other immunosuppressive treatment”**	
Patients ID	Maintenance immunosuppressive treatment
3	Tacrolimus + Mycophenolate
11	Steroids + Tacrolimus
12	Tacrolimus + Mycophenolate
13	Rapamycin + Mycophenolate
14	Steroids + Mycophenolate + Everolimus
25	Tacrolimus + Mycophenolate
29	Steroids + Tacrolimus + Mycophenolate + Leflunomide
30	Steroids + Tacrolimus + Leflunomide
59	Steroids + Tacrolimus

Supplementary Table S7: Univariate Cox model for the kidney failure since the kidney biopsy

Covariates		N	HR	CI	p Value*
**Characteristics at the Lung transplantation**					
**eGFR (ml/min/1.73 m²)**	129-225	24	-	-	-
	112-129	23	1.2	0.5-2.8	0.8
	98.7-112	22	1.3	0.5-3.3	0.6
	<98.7	23	0.3	0.1-1.2	0.08
**Male sex**		55	1.074	0.5-2.1	0.8
**Lung disease**	Tobacco emphysema	19	-	-	-
	Cystic fibrosis	45	1.61	0.6-4.3	0.34
	Pulmonary fibrosis	20	0.94	0.25-3.5	0.93
	Other	16	1.82	0.5-6.3	0.35
**Postoperative hemodialysis**		13	2.7	1.1-6.2	**0.023**
**Acute kidney injury post LT**	KDIGO 0	17	-	-	-
	KDIGO 1	31	0.75	0.2-2.6	0.65
	KDIGO 2	13	1.59	0.4-5.9	0.49
	KDIGO 3	27	2.1	0.7-6.4	0.202
**Per operative cardiopulmonary bypass**		28	1.55	0.7-3.3	0.26
**Kidney parameters at Kidney biopsy**					
**eGFR > 30 ml/min/1.73 m²**		56	0.5	0.3-1.0	0.07
**Age (years)**	< 31.7	25	-	-	-
	31.7 – 47	25	1.1	0.5-2.7	0.8
	47 – 56.5	25	1.6	0.6-3.9	0.4
	> 56.5	25	0.5	0.2-1.6	0.2
**BMI**	< 18.2	22	-	-	-
	18.2 – 20.1	20	1.5	0.5-4.3	0.4
	20.1-22.1	21	2.4	0.8-6.8	0.1
	> 22.1	22	1.0	0.3-3.3	1.0
**Systemic TMA**		15	0.15	0.02-1.1	0.07
**Proteinuria > 3 (g/g)**		13	6.5	2.8-14.9	**<0.001**
**Diabetes**		51	3.2	1.5-7.1	**0.003**
**Arterial hypertention**		60	1.6	0.8-3.3	0.218
**CNI - mTor inhibitor combination**		32	1.2	0.6-2.4	0.7
**Time between LT and KB, per year**		100	1.08	1.0-1.2	**0.01**
**Time between first biological sign of renal injury and KB, per year**		95	1.2	1.1-1.4	**<0.001**
**Histological characteristics**					
**% of glomerulosclerosis**	0-4.4	25	-	-	-
	4.4-13.4	25	2.0	0.5-8.1	0.3
	13.4-43.5	25	4.2	1.1-15.8	**0.03**
	>43.5	25	8.9	2.6-3.5	**<0.001**
**Focal segmental glomerulosclerosis**		29	3.3	1.7-6.6	**<0.001**
**Vascular Fibrous intimal thickening**	Grade 0	13	-	-	-
	Grade 1	30	0.7	0.2-2.3	0.5
	Grade 2-3	49	1.4	0.5-4.1	0.5
**Arteriolar hyalinosis**	Grade 0	13	-	-	-
	Grade 1	28	2.0	0.4-9.5	0.4
	Grade 2-3	57	3.4	0.8-14.6	0.1
**Moderate to severe interstitial fibrosis-tubular atrophy**		61	2.1	0.97-4.5	0.06
**Inflammation in fibrosis**		32	1.8	0.9-3.5	0.10
**Moderate to Severe Acute tubular necrosis**		23	2.19	0.95-5.03	0.06
**Histologic TMA**		24	0.7	0.3-1.6	0.4
**Tubular macrovacuolization**		15	0.8	0.3-2.2	0.6
**Tubular isovolumetric vacuolization**	Grade 0	46	-	-	-
	Grade 1	34	0.9	0.4-2.0	0.8
	Grade 2-3	18	0.9	0.4-2.0	0.7
**Myocyte vacuolization**		9	2.0	0.7-5.6	0.2
**Presence of crystals**		18	0.6	0.2-1.6	0.3
**Diabetic nephropathy**		6	3.9	1.5-10.3	**0.006**

*** p value for Cox test.** N: number of patients. HR: Hazard ratio. CI: Confidence Interval. eGFR: Estimated glomerular filtration rate by CKD EPI 2021. LT: Lung transplantation. BMI: Body Mass index. TMA: Thrombotic microangiopathy. CNI: Calcineurin inhibitor. KB: Kidney biopsy.

Reporting checklist for cohort study.

Based on the STROBE cohort guidelines.

Instructions to authors

Complete this checklist by entering the page numbers from your manuscript where readers will find each of the items listed below.

Your article may not currently address all the items on the checklist. Please modify your text to include the missing information. If you are certain that an item does not apply, please write "n/a" and provide a short explanation.

Upload your completed checklist as an extra file when you submit to a journal.

In your methods section, say that you used the STROBE cohortreporting guidelines, and cite them as:

von Elm E, Altman DG, Egger M, Pocock SJ, Gotzsche PC, Vandenbroucke JP. The Strengthening the Reporting of Observational Studies in Epidemiology (STROBE) Statement: guidelines for reporting observational studies.

		Reporting Item	Page Number
**Title and abstract**			
Title	#1a	Indicate the study's design with a commonly used term in the title or the abstract	2
Abstract	#1b	Provide in the abstract an informative and balanced summary of what was done and what was found	2
**Introduction**			
Background / rationale	#2	Explain the scientific background and rationale for the investigation being reported	3
Objectives	#3	State specific objectives, including any prespecified hypotheses	3

Methods

Study design	#4	Present key elements of study design early in the paper	4
Setting	#5	Describe the setting, locations, and relevant dates, including periods of recruitment, exposure, follow-up, and data collection	4
Eligibility criteria	#6a	Give the eligibility criteria, and the sources and methods of selection of participants. Describe methods of follow-up.	4
Eligibility criteria	#6b	For matched studies, give matching criteria and number of exposed and unexposed	n/a

Variables	#7	Clearly define all outcomes, exposures, predictors, potential confounders, and effect modifiers. Give diagnostic criteria, if applicable	4
Data sources / measurement	#8	For each variable of interest give sources of data and details of methods of assessment (measurement). Describe comparability of assessment methods if there is more than one group. Give information separately for for exposed and unexposed groups if applicable.	4
Bias	#9	Describe any efforts to address potential sources of bias	4
Study size	#10	Explain how the study size was arrived at	4
Quantitative variables	#11	Explain how quantitative variables were handled in the analyses. If applicable, describe which groupings were chosen, and why	4
Statistical methods	#12a	Describe all statistical methods, including those used to control for confounding	5
Statistical methods	#12b	Describe any methods used to examine subgroups and interactions	5
Statistical methods	#12c	Explain how missing data were addressed	5
Statistical methods	#12d	If applicable, explain how loss to follow-up was addressed	n/a
Statistical methods	#12e	Describe any sensitivity analyses	5

**Results**			
Participants	#13a	Report numbers of individuals at each stage of study—eg numbers potentially eligible, examined for eligibility, confirmed eligible, included in the study, completing follow-up, and analysed. Give information separately for for exposed and unexposed groups if applicable.	n/a
Participants	#13b	Give reasons for non-participation at each *stage*	n/a
Participants	#13c	Consider use of a flow diagram	n/a
Descriptive data	#14a	Give characteristics of study participants (eg demographic, clinical, social) and information on exposures and potential confounders. Give information separately for exposed and unexposed groups if applicable.	5-9

Descriptive data	#14b	Indicate number of participants with missing data for each variable of interest	5-9
Descriptive data	#14c	Summarise follow-up time (eg, average and total amount)	8
Outcome data	#15	Report numbers of outcome events or summary measures over time. Give information separately for exposed and unexposed groups if applicable.	5-9
Main results	#16a	Give unadjusted estimates and, if applicable, confounder-adjusted estimates and their precision (eg, 95% confidence interval). Make clear which confounders were adjusted for and why they were included	5-9
Main results	#16b	Report category boundaries when continuous variables were categorized	5-9
Main results	#16c	If relevant, consider translating estimates of relative risk into absolute risk for a meaningful time period	8
Other analyses	#17	Report other analyses done—eg analyses of subgroups and interactions, and sensitivity analyses	5-9
**Discussion**			
Key results	#18	Summarise key results with reference to study objectives	9-11
Limitations	#19	Discuss limitations of the study, taking into account sources of potential bias or imprecision. Discuss both direction and magnitude of any potential bias.	9-11
Interpretation	#20	Give a cautious overall interpretation considering objectives, limitations, multiplicity of analyses, results from similar studies, and other relevant evidence.	9-11
Generalisability	#21	Discuss the generalisability (external validity) of the study results	9-11
**Other Information**			
Funding	#22	Give the source of funding and the role of the funders for the present study and, if applicable, for the original study on which the present article is based	n/a

None The STROBE checklist is distributed under the terms of the Creative Commons Attribution License CC-BY. This checklist can be completed online using https://www.goodreports.org/, a tool made by the EQUATOR Network in collaboration with Penelope.ai
